# Genetic polymorphisms in cGAS-STING-mediated type I interferon innate immune signaling pathway are associated with DLBCL

**DOI:** 10.3389/fimmu.2025.1725218

**Published:** 2025-12-16

**Authors:** Qirui Zhou, Ruinan Jia, Jinlin Chen, Yang Tan, Yuechan Ma, Mengfan Luan, Xue Sheng, Xiao Han, Shuying Li, Fei Lu, Chunyan Ji, Dongmei Wang, Jingjing Ye

**Affiliations:** 1 Department of Hematology, Qilu Hospital of Shandong University, Cheeloo College of Medicine, Shandong University, Jinan, China; 2 Shandong Key Laboratory of Hematological Diseases and Immune Microenvironment, Qilu Hospital of Shandong University, Shandong University, Jinan, China

**Keywords:** DLBCL, innate immune, prognosis, SNPs, treatment response

## Abstract

**Background:**

Diffuse large B cell lymphoma (DLBCL) is a highly heterogeneous lymphoid neoplasm characterized by diverse gene expression profiles and genetic alterations, resulting in substantial variations in clinical features and response to therapy. The cyclic GMP-AMP synthase (cGAS)-stimulator of interferon genes (STING) pathway plays a central role in innate immune response and affects the development of DLBCL. However, the relationship between genetic polymorphisms in genes involved in the cGAS-STING-mediated signaling pathway and their role in DLBCL remains underexplored.

**Methods:**

A total of 147 patients with DLBCL and 247 healthy controls were recruited. Single nucleotide polymorphism (SNP) genotyping was conducted using the MassARRAY platform. We evaluated the associations between the selected SNPs and DLBCL susceptibility, clinical features, and survival.

**Results:**

In our study, TREX1 rs11797 and CXCL10 rs4508917 showed significant association with DLBCL susceptibility. IFNB1 rs1051922 was correlated with white blood cell (WBC) and monocyte count at diagnosis. TREX1 rs11797 and IFNB1 rs1051922 were associated with chemotherapy response in DLBCL. Moreover, PRMT1 rs975484 and CXCL10 rs8878 were associated with the overall survival of patients with DLBCL. Notably, PRMT1 rs975484 was also correlated with hemoglobin (HGB) level, and may serve as an independent favorable prognostic factor in DLBCL.

**Conclusions:**

Our findings suggest that SNPs involved in cGAS-STING-mediated type I interferon pathway may influence DLBCL susceptibility, treatment response, and prognosis, highlighting their potential as biomarkers for risk stratification and for guiding individualized disease monitoring.

## Introduction

Diffuse large B-cell lymphoma (DLBCL) is a lymphoid neoplasm characterized by significant molecular and clinical heterogeneity (1). Although R-CHOP (rituximab, cyclophosphamide, doxorubicin, vincristine, and prednisone) immunochemotherapy cures more than 60% of patients, the remainder do not respond and have a dismal prognosis (2). Consequently, research has focused on uncovering the molecular determinants of treatment failure. Genomic research has advanced our understanding of prognostic biomarkers in DLBCL, enabling more precise molecular classification and outcome prediction (3).

The cyclic GMP-AMP synthase (cGAS)-stimulator of interferon genes (STING) pathway, encoded by transmembrane protein 173 (TMEM173), represents a central innate immune mechanism linking cytosolic DNA recognition to antitumor defense (4, 5). Upon DNA binding, cGAS produces the second messenger 2’3’-cGAMP, which activates ER-localized STING. This triggers TBK1-IRF3 signaling and the production of type I interferons. A key downstream effector, the chemokine CXCL10, recruits T and NK cells to promote tumor infiltration and influence treatment outcomes (6–10). To prevent excessive activation, negative regulators like three-prime repair exonuclease 1 (TREX1) degrade cytosolic DNA, while protein arginine methyltransferase 1 (PRMT1) inhibits cGAS dimerization (11–15). This pathway has also been implicated in DLBCL. STING up-regulation suppresses DLBCL cell growth by inducing cell death (16), and a meta-analysis identified it as a low-risk gene in DLBCL (17). Moreover, a prospective cohort study showed that high serum CXCL10 levels were associated with increased tumor burden, suggesting its potential utility as a biomarker (18). Thus, the cGAS-STING-mediated type I interferon pathway may play an important role in pathogenesis and development of DLBCL.

Single nucleotide polymorphisms (SNPs), as the most prevalent genetic variants, can modulate gene regulation at multiple levels and thereby influence disease susceptibility and clinical outcomes (19). SNP-based risk scores have been shown to predict clinical outcomes and survival in cancer patients (3, 20), and increasing evidence suggests that SNPs in immune signaling pathways play crucial roles in prognosis of various tumors (21, 22). In diverse cancers, epigenetic inactivation of STING contributes to tumor immune evasion (23, 24), and may be a potential new target to improve DLBCL prognosis from an immunological perspective (25–27). Variants in STING1 have been linked to cervical cancer development (28), while in patients with metastatic colorectal cancer treated with cetuximab-based first-line therapy, IFNB1 rs1051922 correlates with progression-free survival (29). Moreover, polymorphisms in cGAS rs72960018, cGAS rs9352000 and STING rs13153461 have been associated with increased colorectal cancer risk (30).

However, the distribution and clinical relevance of SNPs within the cGAS-STING-mediated type I interferon pathway remain largely unexplored in patients with DLBCL. In this study, we genotyped a set of SNPs in 7 genes of this pathway by 147 DLBCL cases and 247 healthy controls, and evaluated their association with lymphoma risk, clinical features, and patient survival.

## Materials and methods

### Study participants

For genetic polymorphisms detection, 150 patients with newly diagnosed DLBCL according to the World Health Organization classification (31) were recruited from Qilu Hospital of Shandong University from August 2020 to June 2025. 250 healthy controls were recruited from the Health Examination Center of Qilu Hospital, who were matched to the DLBCL patients by age, sex, and ethnicity, and confirmed to have no history of cancer, autoimmune diseases, or chronic viral infections. Peripheral blood was collected from newly diagnosed patients before initial therapy and from the control group on the day of physical examination. All specimens were collected in anticoagulant tubes and stored at -80°C. After quality control procedures, 147 DLBCL cases and 247 healthy controls were successfully genotyped and included in the final analysis. Treatment response was evaluated by blinded assessors using the Lugano Response Criteria, with a central imaging review. All participants provided written informed consent before enrollment, and the study was approved by Medical Ethics Committee of Qilu Hospital of Shandong University (No. KYLL-2024(ZM)-161).

### DNA extraction and SNP genotyping

Genomic DNA was extracted from peripheral blood of DLBCL patients and healthy controls using a DNA extraction kit (Tiangen Biotech, Beijing, China) according to the manufacturer’s instructions. SNP genotyping was conducted using the MassARRAY platform (BGI Tech, Beijing, China). Primers were designed using Assay Designer 4.0 software(Agena Bioscience, San Diego, CA, USA). The procedure consisted of polymerase chain reaction (PCR) amplification of target fragments, shrimp alkaline phosphatase (SAP) treatment, single-base extension reactions, and resin purification. Matrix-assisted laser desorption/ionization time-of-flight (MALDI-TOF) mass spectrometry was then performed for allele detection, and genotype data were analyzed using TYPER 4.0 software (Agena Bioscience, San Diego, CA, USA) according to the manufacturer’s protocol.

### Statistical analysis

For each SNP, genotype frequencies in the control group were examined. Minor allele frequency (MAF) was calculated from allele counts, and Hardy-Weinberg equilibrium (HWE) was assessed using Pearson’s χ² goodness-of-fit test. For each SNP, the minor allele was pre-specified as the effect allele. All genetic models were parameterized with respect to this effect allele. Three genetic models were used to analyze the genotype data: codominant, dominant and recessive. The associations of SNP genotypes and allele frequencies with DLBCL susceptibility and clinical characteristics were initially assessed using the chi-squared test or Fisher’s exact test. Subsequently, multivariate binary logistic regression was used to calculate odds ratios (ORs) and 95% confidence intervals (CIs), adjusting for age and sex. False discovery rate (FDR) correction was applied separately within three endpoint‐specific families: disease susceptibility, baseline laboratory traits, and chemotherapy response. Within each family, all SNPs evaluated under different genetic inheritance models (dominant, recessive, and codominant) were jointly corrected. Because one SNP (CXCL10 rs8878) exhibited an absent genotype category, its association analysis was performed using Firth bias-reduced logistic regression. The corresponding per-genotype counts and statistical results were reported separately in **Supplementary Table 2**. Kaplan-Meier curves were used to estimate overall survival (OS), and Cox regression analysis was used to evaluate prognostic factors of DLBCL. The proportional‐hazards assumption was evaluated by Schoenfeld residuals. Statistical analyses were performed in IBM SPSS Statistics version 27.0 (IBM Corp., Armonk, NY, USA), R (version 4.4.1) and GraphPad Prism 10 (GraphPad Software, San Diego, CA, USA). A P value < 0.05 or a false discovery rate (FDR) q value < 0.05 was considered statistically significant.

## Results

### SNP selection and study populations

The selected SNPs are listed in **Table 1**. The candidate SNPs were reported in previous studies to be significantly associated with specific disease or immune-related phenotypes. Moreover, SNPs located in regulatory regions such as promoters or enhancers, and those that may affect transcription factor binding sites were preferentially included. The cGAS and TMEM173, as core molecules of the cGAS-STING pathway with limited research on their SNPs, were also selected. Variants were further filtered by call rate. HWE and MAF were used for the initial screening of eight candidate SNPs. SNPs deviating from HWE (p < 0.05) or with a MAF< 0.05 were excluded from further analyses. The characteristics of 147 DLBCL patients and 247 healthy controls are shown in **Table 2**, along with routine blood counts, risk stratification, and treatment response data.

**Table 1:** Selected genes and SNPs.

Gene	SNP	Variant	Minor allele	MAF	HWE(P-value)
TREX1	rs11797	C>T	T	0.3178	0.2465525
cGAS	rs72960018*	G>A	A	0.0223	0.7203982
TMEM173	rs13153461*	G>A	A	0.4575	0.0332724
IFNB1	rs1051922	G>A	A	0.3340	0.1194011
PRMT1	rs975484	C>G	G	0.2085	0.9194927
IRF3	rs2304205	A>C	C	0.1903	0.3956891
CXCL10	rs4508917	G>A	A	0.4312	0.5894708
	rs8878	G>A	A	0.0526	0.6869627

*SNP was excluded in further analysis.

**Table 2:** Demographic and clinical characteristics of DLBCL patients and healthy controls.

Characteristic	Case N(%)	Control N(%)
Age(years,median,range)	58(19,84)	58(20,88)
<60	78(53.1)	136(55.1)
≥ 60	69(46.9)	111(44.9)
Gender		
Male	81(55.1)	130(52.6)
Female	66(44.9)	117(47.4)
WBC		
Median(×10^9)	6.01	NA
≤9.5	129(87.8)	NA
>9.5	18(12.2)	NA
HGB		
Median(g/L)	124	NA
≥90	135(91.8)	NA
<90	12(8.2)	NA
PLT		
Median(×10^9/L)	235	NA
≥100	138(93.9)	NA
<100	9(6.1)	NA
B symptoms		
No	106(72.1)	NA
Yes	41(27.9)	NA
Hans Type		
GCB	45(30.6)	NA
Non-GCB	68(46.3)	NA
Unknown	34(23.1)	NA
IPI score risk groups		
Low risk	43(29.3)	NA
Medium to low risk	34(23.1)	NA
Medium to high risk	29(19.7)	NA
High risk	41(27.9)	NA
Marrow involved		
No	137(93.2)	NA
Yes	10(6.8)	NA
Treatment Response*		
CR	75(62.0)	NA
PR	17(14.0)	NA
SD	9(7.4)	NA
PD	20(16.5)	NA

*patients evaluated after 4 cycles of treatment (n=121)

### CXCL10 rs4508917 and TREX1 rs11797 are associated with DLBCL susceptibility

We used three genetic models (codominant, dominant and recessive) to analyze the associations of the selected SNPs with DLBCL susceptibility (**Table 3**). Preliminary screening with the chi-square or Fisher’s exact test showed that the CXCL10 rs4508917 and TREX1 rs11797 were significantly correlated with DLBCL susceptibility under the codominant and dominant models (p < 0.05), and IFNB1 rs1051922 was correlated with DLBCL susceptibility under the recessive model (p < 0.05). After adjusting for sex and age, and applying FDR correction for multiple testing, under the codominant model, the AA genotype of CXCL10 rs4508917 was found to be a risk factor for DLBCL susceptibility compared to the GG genotype, with the effect magnitude being moderate (p = 0.046). In contrast, under the codominant model of TREX1 rs11797, the CT genotype was associated with a reduced risk of DLBCL compared with the CC genotype with a moderate degree (p = 0.046) (**Supplementary Figure 1A**).

**Table 3:** Association between SNPs and DLBCL susceptibility.

Gene	SNP	Model	Genotype	Control (n)	DLBCL case(n)	χ2 test P value	OR (95%CI)	Adjusted P value
IFNB1	rs1051922	Recessive	AG+GG	214	137	0.043		
			AA	33	10		0.470 (0.224, 0.986)	0.064
CXCL10	rs4508917	Co-dominant	GG	82	33	0.036		
			AG	117	73		1.516 (0.918, 2.506)	0.121
			AA	48	41		2.098 (1.172, 3.755)	0.046
		Dominant	GG	82	33	0.023		
			AA+AG	165	114		1.702 (1.063, 2.725)	0.053
TREX1	rs11797	Co-dominant	CC	111	83	0.011		
			CT	115	46		0.541 (0.346, 0.845)	0.046
			TT	21	18		1.250 (0.617, 2.532)	0.535
		Dominant	CC	111	83	0.027		
			TT+CT	136	64		0.633 (0.419, 0.956)	0.053

SNP, single nucleotide polymorphisms; DLBCL, Diffuse large B cell lymphoma; OR, odds ratio; CI, confidence interval.

### Associations between SNPs and baseline clinical characteristics of DLBCL patients

To assess the potential impact of genetic variation on hematologic parameters, we analyzed the associations of selected SNPs with white blood cell (WBC), platelet (PLT) counts and hemoglobin (HGB) in DLBCL patients using the χ² test or Fisher’s exact test.

The high-WBC group was defined as patients with count>9.5×10^9^/L, and we further examined the associations of SNPs with monocyte and lymphocyte counts. As shown in **Table 4**, χ2 test suggested that IFNB1 rs1051922 was linked to WBC count (p < 0.05) and monocyte count (p < 0.05) under the dominant model. After controlling for age and sex and correcting for multiple testing using FDR, carriers with the AG/AA genotype of IFNB1 rs1051922 under the dominant model showed a significantly reduced tendency toward leukocytosis with a large effect size (p = 0.039), indicating a protective effect on white blood cell homeostasis. Similarly, individuals with the same genotype were more likely to maintain normal monocyte counts with a medium degree (p = 0.039), further supporting its protective role in peripheral immune regulation (**Supplementary Figure 1C**).

**Table 4:** Association between SNPs and the baseline data of DLBCL patients.

Gene	SNP	Model	Genotype	WBC≤ 9.5*10^9/L(n)	WBC> 9.5*10^9/L(n)	χ2 test P value	OR (95%CI)	Adjusted P value
IFNB1	rs1051922	Dominant	GG	55	13	0.018		
			AA+AG	74	5		0.288 (0.097, 0.856)	0.039

Gene	SNP	Model	Genotype	Normal monocyte count(n)	Abnormal monocyte count(n)	χ2 test *P* value	OR(95%CI)	Adjusted *P* value
IFNB1	rs1051922	Dominant	GG	42	26	0.026		
			AA+AG	62	17		0.443 (0.214, 0.920)	0.039

Gene	SNP	Model	Genotype	HGB≥90g/L(n)	HGB<90g/L(n)	Fisher’s exact test *P* value	OR (95%CI)	Adjusted P value
PRMT1	rs975484	Codominant	CC	91	5	0.029		
			CG	41	5		2.253 (0.613, 8.285)	0.221
			GG	3	2		10.103 (1.268, 80.529)	0.039

SNP, single nucleotide polymorphisms; OR, odds ratio; CI, confidence interval.

For the analysis of peripheral blood PLT counts, patients were stratified into a high-PLT group (≥100 × 10^9^/L) and a low-PLT group (<100 × 10^9^/L). HGB levels were categorized as high (≥ 90g/L) or low (< 90g/L). Fisher’s exact test revealed that rs975484 was significantly associated with HGB level under the codominant model (p < 0.05). After adjusting for age and sex with FDR correction, compared with the CC genotype, carriers of the GG genotype in rs975484 promoted low HGB levels, with the effect magnitude being large (p = 0.039), whereas the CG genotype did not show a significant association (**Supplementary Figure 1B**). No SNP showed a significant association with PLT level in patients with DLBCL (p > 0.05).

### TREX1 rs11797, IFNB1 rs1051922 and DLBCL treatment sensitivity

We evaluated the associations between SNPs and chemotherapy sensitivity in DLBCL patients, with treatment response assessed in those who had completed at least four cycles of chemotherapy (n=121), as this is generally considered the minimum exposure required for reliable response evaluation (**Table 5**). In preliminary analyses using the χ² test, the TREX1 rs11797 and IFNB1 rs1051922 variants under the dominant model showed a potential association with treatment response (p < 0.05). Given the heterogeneity of treatment regimens, first-line systemic therapy was classified into two groups: R-CHOP–based immunochemotherapy and other regimens (R-DA-EPOCH, R-EPOCH, OR2-EDOCH, R-CHOP plus BTKi, and POLA-R-CHP), which are generally used in selected clinical contexts. To account for treatment heterogeneity, first-line regimen category (R-CHOP–based *vs* other regimens) was included as a covariate in the multivariable logistic regression model evaluating factors associated with complete response (CR).

**Table 5:** TREX1 rs11797, IFNB1 rs1051922 were associated with DLBCL chemotherapy response.

Gene	SNP	Model	Genotype	No CR (n)	CR (n)	χ2 test P value	OR(95%CI)	Adjusted P value
TREX1	rs11797	Dominant	CC	32	36	0.020		
			CT+TT	14	39		2.604 (1.140, 5.947)	0.046

Gene	SNP	Model	Genotype	No CR (n)	CR (n)	χ2 test P value	OR(95%CI)	Adjusted P value
IFNB1	rs1051922	Dominant	GG	26	28	0.039		
			AG+AA	20	47		2.155 (1.005, 4.618)	0.048

SNP, single nucleotide polymorphisms; No CR, non-remission; CR, complete remission; OR, odds ratio; CI, confidence interval.

After adjustment for sex, age, regimen category, and multiple testing (FDR correction), in the dominant model, possession of the TREX1 rs11797 CT/TT genotype conferred a significantly better response to chemotherapy, demonstrating a moderate effect size on treatment outcome. (p = 0.046) (**Supplementary Figure 1A**). Similarly, the AG/AA genotype of IFNB1 rs1051922 in the dominant model moderately led to a higher likelihood of achieving complete remission (CR) (p = 0.048) (**Supplementary Figure 1C**).

### PRMT1 rs975484 and CXCL10 rs8878 are associated with the OS in DLBCL

**Figure 1:**
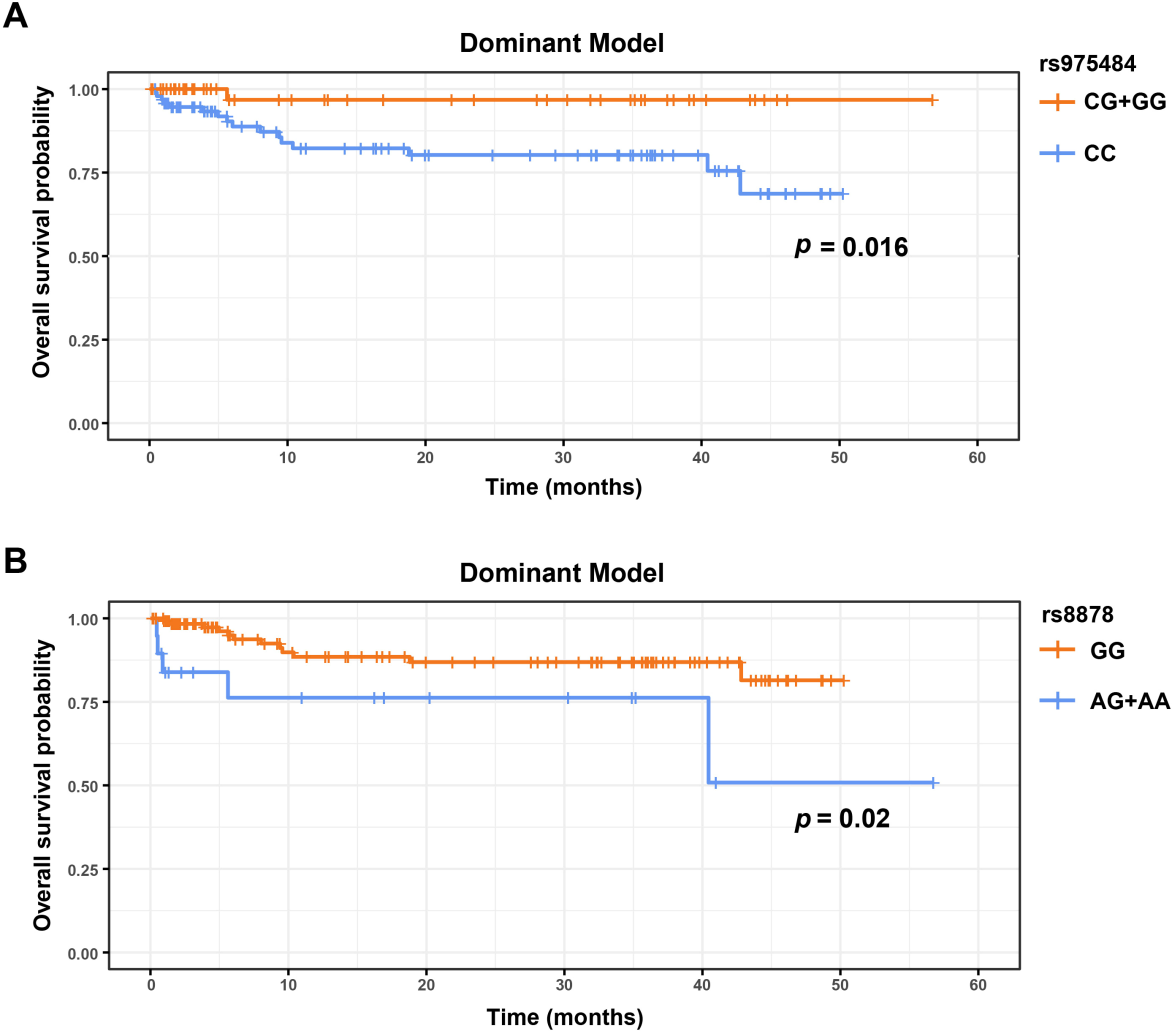
**(A)** The overall survival of DLBCL patients with CG, GG and CC genotypes in PRMT1 rs975484 dominant model. **(B)** The overall survival of DLBCL patients with AG, AA and GG genotypes in CXCL10 rs8878 dominant model.

A total of 17 deaths were recorded among 147 patients. The median follow-up duration was 18.4 months (95% CI, 12.6–30.3), and the interquartile range (IQR) was 3.7–36.6 months. Three genetic models were applied to evaluate the associations of SNPs with overall survival (OS). Kaplan-Meier analysis revealed that the rs975484 variant in PRMT1 and rs8878 variant in CXCL10 were significantly associated with prognosis under the dominant model (p<0.05). Under the dominant model of rs975484 in PRMT1 (**Figure 1A**), patients carrying the CG/GG genotype had significantly longer OS than those with the CC genotype (log-rank p = 0.016). Under the dominant model of rs8878 in CXCL10 (**Figure 1B**), patients with the GG genotype had better OS compared to those with the AA/AG genotypes (log-rank p = 0.020). Because no individuals with the rs8878 AA genotype were identified among cases, dominant-model analyses (AG/AA *vs* GG) were driven mainly by AG carriers. Kaplan–Meier and Cox results therefore reflect the AG effect, and interpretation should consider the limited statistical power due to rare AA counts.

Patients with PLT count <100 × 10 ^9^/L had shorter OS than those with PLT ≥ 100 × 10 ^9^/L (p < 0.001). In addition, the presence of B symptoms was correlated with shorter OS relative to patients without B symptoms (p = 0.018).

### PRMT1 rs975484 is associated with the outcome in DLBCL

**Figure 2:**
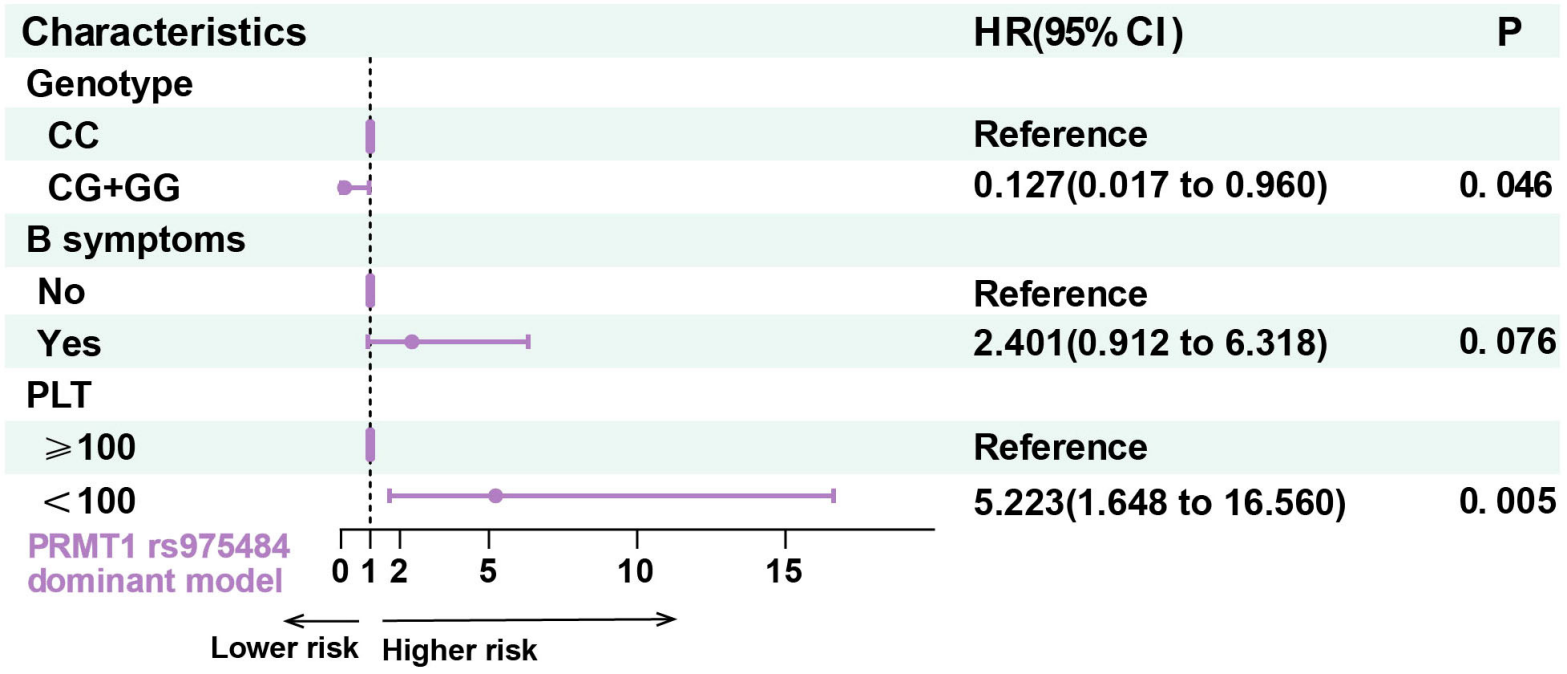
The impact of PRMT1 rs975484 on the outcome of DLBCL patients.

Following univariate analysis, the genotypes of PRMT1 rs975484, CXCL10 rs8878 and relevant clinical variables (including PLT count and B symptoms at diagnosis) were entered into a multivariate Cox proportional hazards regression model to assess their prognostic value. Multivariate Cox regression analysis showed that in the dominant model, the CG/GG genotype of PRMT1 rs975484 retained independent prognostic value, identifying patients with a more favorable clinical outcome in DLBCL (HR = 0.127, 95%CI = 0.017-0.960, p = 0.046) (**Figure 2**). Conversely, the CXCL10 rs8878 AG/AA genotype showed no significant association in Firth Cox regression analysis and was not established as an independent prognostic risk factor in DLBCL.

The proportional-hazards (PH) assumption underlying the Cox models was evaluated using Schoenfeld residuals. Neither individual covariates nor the global model demonstrated significant deviation from the PH assumption (GLOBAL χ²=3.392, df=3, p=0.34).

### CXCL10, TREX1 and PRMT1 are highly expressed in DLBCL patients

**Figure 3:**
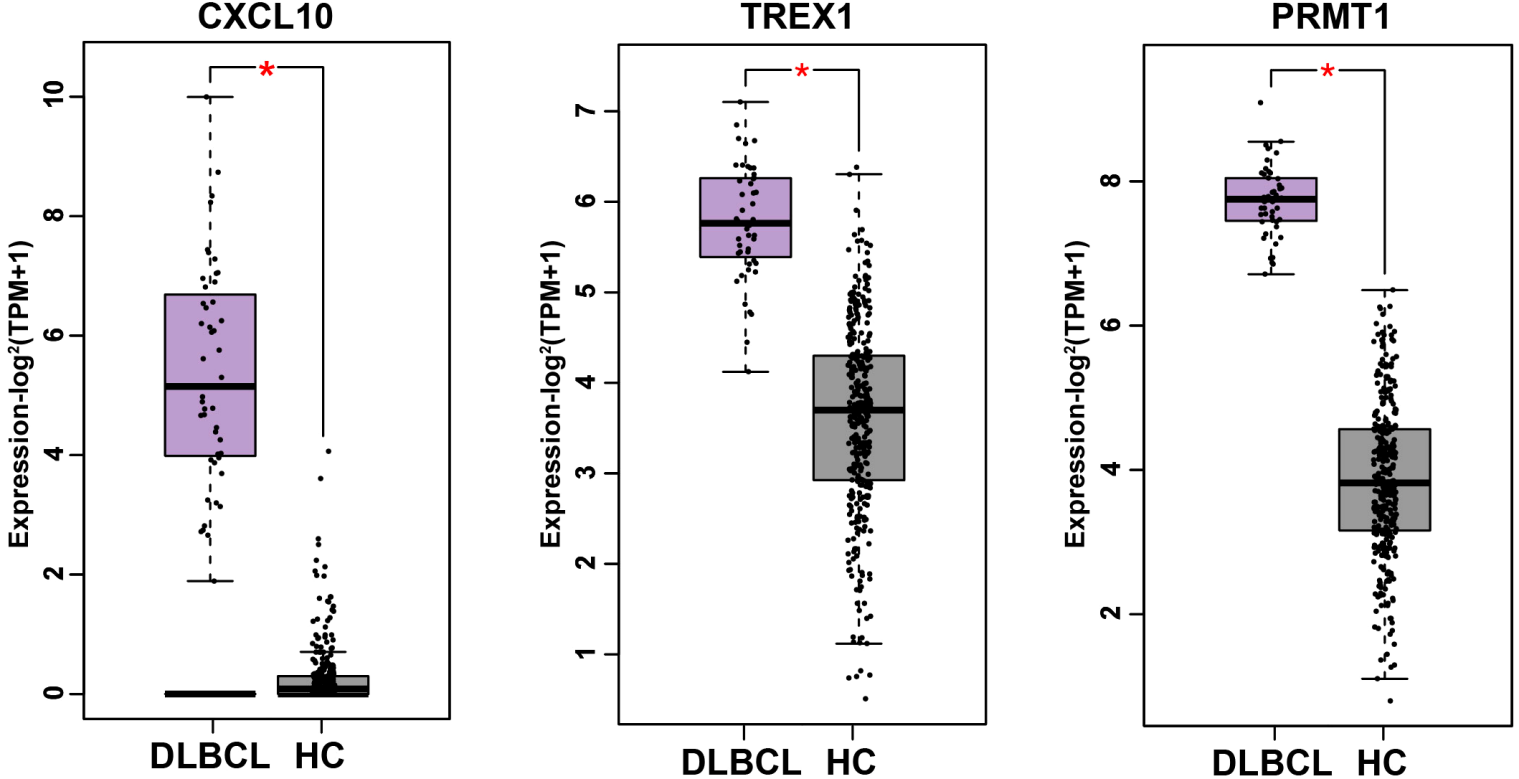
mRNA expression of CXCL10,TREX1 and PRMT1 in DLBCL patients (TCGA) and healthy controls (GTEx) (47 in DLBCL group and 337 in healthy control (HC) group). *P < 0.05.

The expression levels of CXCL10, TREX1 and PRMT1 in DLBCL patients versus healthy controls were analyzed using the Gene Expression Profiling Interactive Analysis 2 (GEPIA2) tool. As shown in **Figure 3**, all three genes were significantly upregulated in DLBCL samples compared with controls (p < 0.05). To investigate the potential regulatory functions of the SNPs, we inquired public expression quantitative trait locus (eQTL) databases. GTEx v10 data reveal that both rs4508917 and rs8878 act as significant cis-eQTLs for CXCL10 in normal tissues. Furthermore, RegulomeDB assign a high-confidence regulatory score (Rank = 1f; Score = 0.55436) to rs11797 in lymphoblastoid cells, suggesting its role in modulating TREX1 expression. Based on these evidences, we hypothesize that these SNPs may contribute to DLBCL pathogenesis by influencing gene expression. However, direct validation of their functional consequences, such as through independent eQTL analyses in DLBCL cohorts, is warranted.

## Discussion

**Figure 4:**
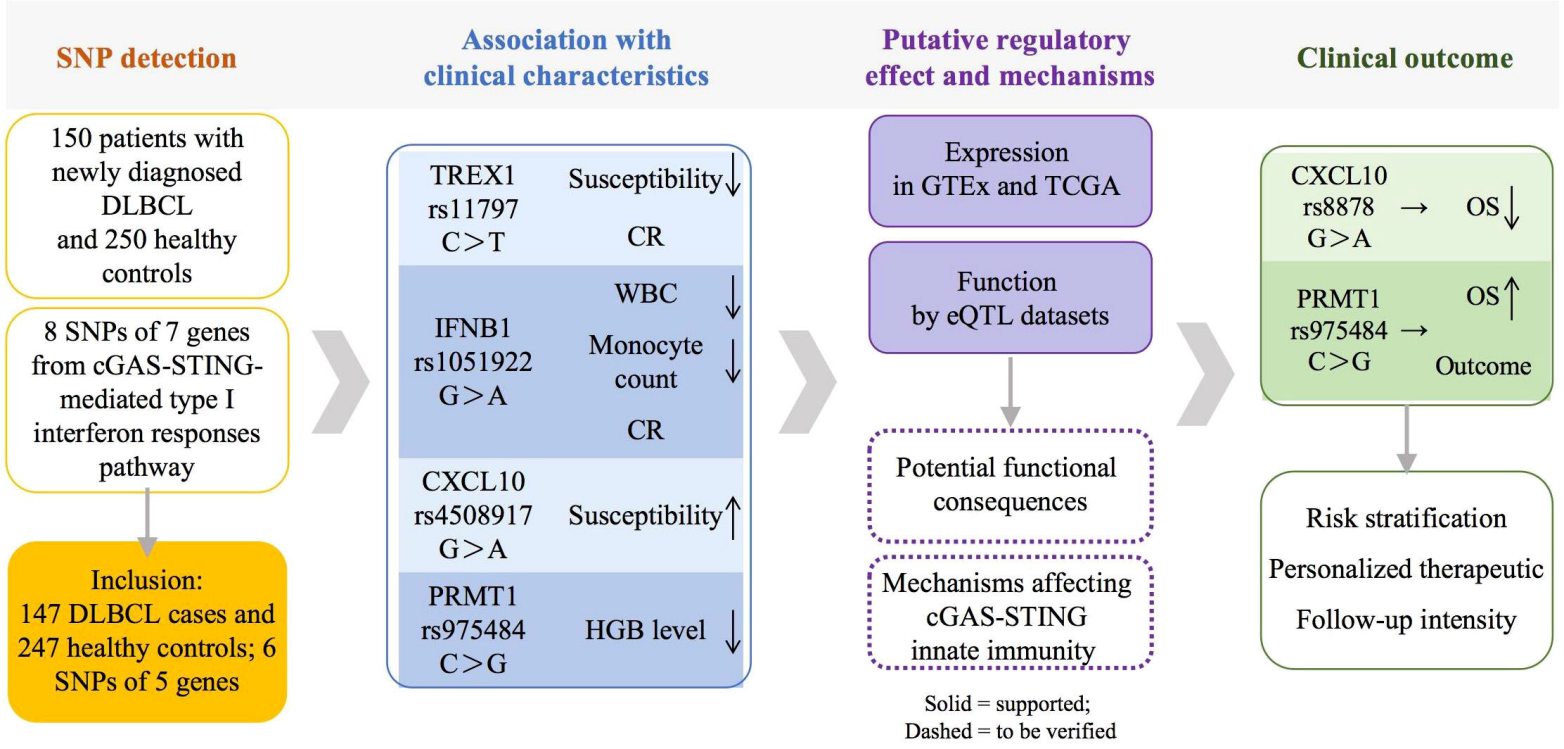
Overview of the SNPs analysis and the translational pathway.

In this study, we focused on SNPs in molecules of the cGAS-STING-mediated type I interferon responses pathway, which were genotyped using the MassARRAY platform in 147 DLBCL patients and 247 healthy controls. In our study, TREX1 rs11797 was related to DLBCL susceptibility and treatment response. IFNB1 rs1051922 was correlated with WBC and monocyte counts as well as treatment response. CXCL10 rs4508917 was associated with DLBCL susceptibility, while rs8878 was linked to OS. Notably, PRMT1 rs975484 was associated with HGB levels and OS by Kaplan-Meier analysis, and it remained an independent predictor of OS in multivariate Cox regression (**Figure 4**), implying that rs975484 may serve as a favorable prognostic biomarker in DLBCL. Our findings suggest that genetic variants within this pathway may contribute to DLBCL susceptibility, influence response to induction chemotherapy, and affect overall prognosis.

Multiple reports have suggested that TREX1 contributes to the therapy resistance and targeting TREX1 induces an innate immune response and resensitizes SCLC cells to chemotherapy (32). Our study identified an association between TREX1 rs11797 and disease susceptibility, which expands the understanding of TREX1 beyond its well-recognized role in therapy response. In the codominant model, we observed that the CT genotype of TREX1 rs11797 conferred a protective effect against disease susceptibility, whereas the TT genotype did not show a significant association. This discrepancy may be partly explained by the relatively small number of individuals with the TT genotype, leading to limited statistical power. In addition, it is possible that the effect of this variant does not follow a simple additive pattern but rather reflects the heterozygote advantage, in which the presence of both alleles provides a favorable balance of gene function. In a study investigating diversity at human leukocyte antigen (HLA) loci in colorectal cancer (CRC), it was reported that individuals carrying heterozygous genotypes at all three class I genes exhibited a reduced risk of CRC, providing evidence for a heterozygote advantage at these loci (33). Moreover, our data suggest that the CT/TT genotype of TREX1 rs11797 was associated with an improved likelihood of achieving CR under the dominant model.

PRMT1, a ubiquitously expressed enzyme, regulates a wide range of molecular and cellular processes, including transcriptional regulation, DNA damage response, mRNA translation, cell division, apoptosis, and signal transduction (34, 35). Infantino et al. identified PRMT1 as a central regulator of humoral immunity, revealing its role in B-cell activation, proliferation, and differentiation (36). Several studies have reported that high PRMT1 expression is associated with prognosis in gastric and breast cancers (37, 38). In our study, we also demonstrated the prognostic relevance of PRMT1. Kaplan–Meier survival analysis revealed that patients carrying the CG/GG genotype had significantly better OS compared with those harboring the CC genotype. Consistently, multivariate Cox regression analysis showed that the CG/GG genotype under the dominant model was independently associated with improved prognosis. Our findings suggest that PRMT1 genetic variants may modulate its oncogenic function or immune regulatory effects, thereby contributing to improved survival in DLBCL. Apart from its prognostic significance, PRMT1 may also influence hematologic parameters. Previous studies have reported that PRMT1 promotes hemoglobin synthesis and erythroid maturation through the induction of glycophorin A and activation of lineage-specific transcription factors (39). Consistently, our study revealed that carriers of the GG genotype in rs975484 were more likely to have lower hemoglobin levels, suggesting that genetic variation in PRMT1 may affect erythropoiesis in DLBCL. Further investigations are needed to elucidate how these polymorphisms affect PRMT1 activity and to evaluate their potential as prognostic biomarkers.

Previous studies have demonstrated that genetic variation in the cGAS-STING -mediated type I interferon pathway may influence the response to cetuximab in metastatic colorectal cancer. In particular, patients with metastatic colorectal cancer carrying IFNB1 rs1051922 G/A and A/A allele showed a significantly shorter PFS than carriers of G/G allele (29). In contrast, no association with survival was observed in our DLBCL cohort, while the AG/AA genotype of IFNB1 rs1051922, under the dominant model, was associated with a higher likelihood of achieving CR following chemotherapy, suggesting a potential role of this variant in treatment response rather than long-term survival. In addition, we found that IFNB1 rs1051922 AG/AA genotype was associated with a reduced risk of elevated WBC count under the dominant model, and seemed to be a protective factor for abnormal peripheral blood monocyte count, suggesting a potential regulatory role in peripheral immune balance. Consistent with previous findings showing that a single IFNβ-1b injection induces rapid changes in leukocyte distribution in healthy individuals (40), our results suggest that genetic variation in IFNB1 may modulate this pathway. Clinical studies of IFN-β therapy have reported early hematologic abnormalities, including lymphopenia and leukopenia, reflecting its immunomodulatory effects on hematopoiesis (41). Taken together, these findings imply that IFNB1 polymorphisms may influence leukocyte homeostasis and inflammatory responses through altered interferon signaling, potentially contributing to immune regulation and disease.

CXCL10, also known as interferon-inducible T-cell alpha chemoattractant or interferon-γ-inducible protein 10, is a small cytokine of the CXC chemokine family. CXCL10 regulates the migration and spatial localization of immune cells within tissues, thereby playing a pivotal role in the innate immune response during infection (7). Evidence from both papillary thyroid carcinoma and triple-negative breast cancer suggests that elevated CXCL10 expression consistently correlates with improved prognosis, likely reflecting its role in promoting antitumor immune responses across distinct tumor types (42, 43). Moreover, the correlation of CXCL10 with immune checkpoint blockade (ICB)-related genes has been reported not only in breast cancer but also consistently across 32 other malignancies, including DLBCL (44), highlighting its broad involvement in tumor immune regulation. Consistent with these findings, in our study, under the dominant model of rs8878 in CXCL10, patients with the GG genotype had better OS compared to those with the AA/AG genotype. Although a number of studies have highlighted the impact of CXCL10 on prognosis in various malignancies, only a few have examined their contribution to disease susceptibility. In our study, in the codominant model, carriers with the AA genotype of CXCL10 rs4508917 showed a tendency toward increased susceptibility to DLBCL, providing a new evidence of the impact of CXCL10 on DLBCL.

Nevertheless, these findings from genotype–phenotype association analyses are preliminary, and the precise molecular mechanisms underlying the cGAS-STING-mediated type I interferon pathway mediated by the identified SNPs remain to be elucidated. Future investigations will focus on clarifying how these SNPs contribute to the pathogenesis and progression of DLBCL. In addition, there are several limitations to the present study. First, modest cohort size and the limited number of events possibly reduced the stability and precision of the multivariable Cox estimates, which caused several adjusted p-values sit closing to 0.05 and a Cox estimate exhibiting wide confidence intervals. Second, the potential confounding factor of subtle population structure may introduce bias even in single-center genetic studies, and the requisite ancestry principal components (PCs) could not be derived due to insufficient genome-wide markers. As a result, minor population stratification may not be fully adjusted for. Finally, an additional methodological limitation should be acknowledged. χ²-based prefiltering may influence false-discovery estimation and risk omitting SNPs that become significant only after covariate adjustment. Therefore, these findings should be viewed as exploratory, which require validation in independent cohorts with broader genomic coverage and functional experiments to verify how these SNPs influence DLCBL prognosis.

## Conclusion

This study identifies genetic variants in the cGAS-STING pathway as significant determinants of susceptibility, treatment response, and survival in Chinese patients with DLBCL. These variants of TREX1, IFNB1, CXCL10 and PRMT1 could serve as practical clinical biomarkers for risk stratification at diagnosis, which could guide personalized therapeutic and follow-up intensity, and grounds this proposal in our specific statistical findings (ORs/HRs and 95% CIs) (**Figure 4**).

## Data Availability

The original contributions presented in the study are included in the article/**Supplementary Files**. Further inquiries can be directed to the corresponding authors.
