# Correction: Genetic polymorphisms in cGAS-STING-mediated type I interferon innate immune signaling pathway are associated with DLBCL

**DOI:** 10.3389/fimmu.2025.1773500

**Published:** 2026-01-05

**Authors:** Qirui Zhou, Ruinan Jia, Jinlin Chen, Yang Tan, Yuechan Ma, Mengfan Luan, Xue Sheng, Xiao Han, Shuying Li, Fei Lu, Chunyan Ji, Dongmei Wang, Jingjing Ye

**Affiliations:** 1 Department of Hematology, Qilu Hospital of Shandong University, Cheeloo College of Medicine, Shandong University, Jinan, China; 2 Shandong Key Laboratory of Hematological Diseases and Immune Microenvironment, Qilu Hospital of Shandong University, Shandong University, Jinan, China

**Keywords:** DLBCL, innate immune, prognosis, SNPs, treatment response

Affiliation “Department of Hematology, Qilu Hospital of Shandong University, Cheeloo College of Medicine, Shandong University, Jinan, China” was omitted for author Dongmei Wang. This affiliation has now been added for author Dongmei Wang.

Affiliation “Shandong Key Laboratory of Hematological Diseases and Immune Microenvironment, Qilu Hospital of Shandong University, Shandong University, Jinan, China” was omitted for author Fei Lu. This affiliation has now been added for author Fei Lu.

The original version of this article has been updated.

