# Supplementary material for: Genetic polymorphisms in cGAS-STING-mediated type I interferon innate immune signaling pathway are associated with DLBCL
Source: Front Immunol. 2025 Dec 16;16:1725218. doi: 10.3389/fimmu.2025.1725218 (PMC12747902; doi:10.3389/fimmu.2025.1725218)

**Supplementary Files**

**Supplementary Figure 1.** The forest plots for OR (95% CI) of TREX1 rs11797 (A), PRMT1 rs975484 (B), IFNB1 rs1051922 (C).


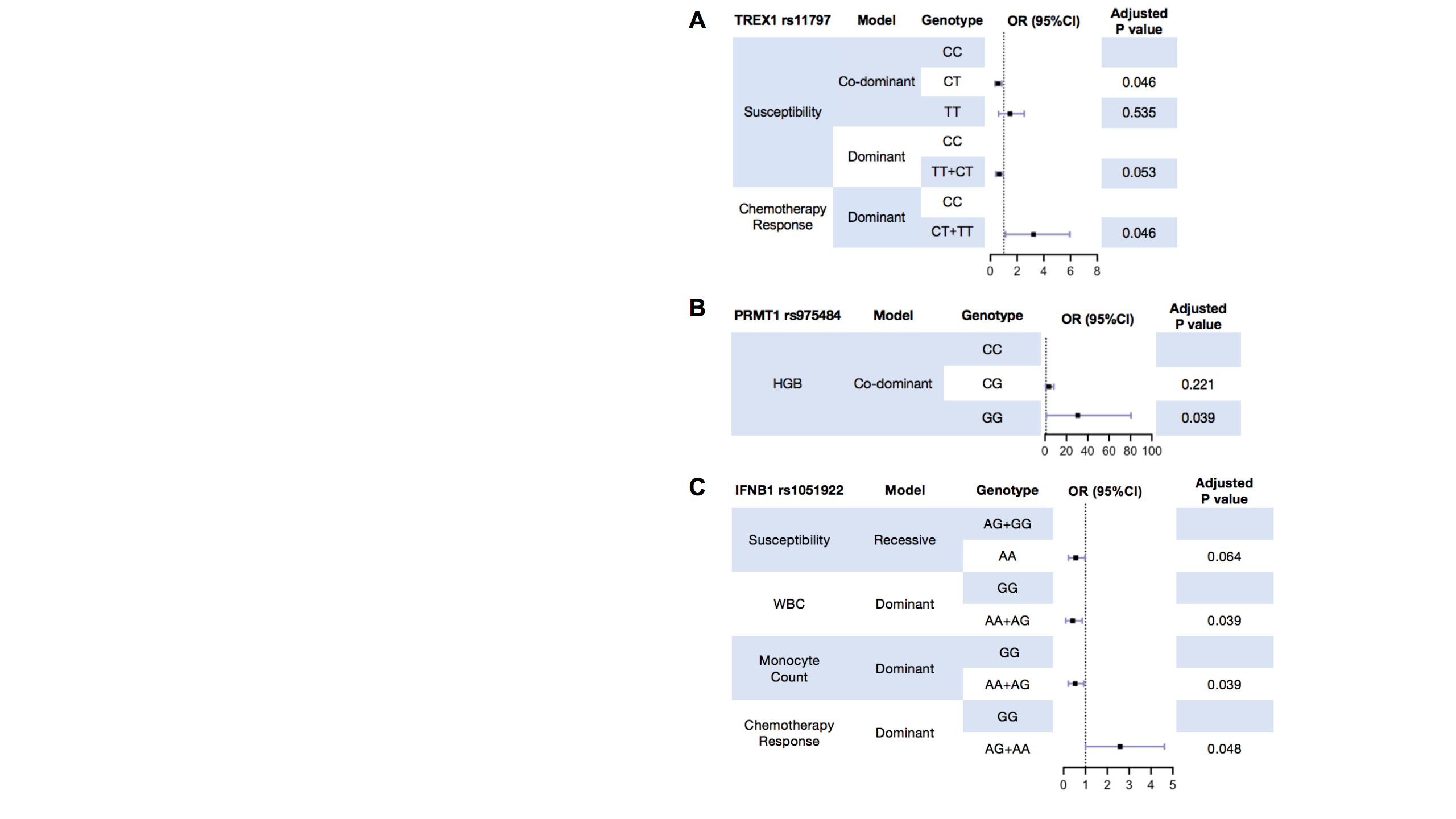

Supplement: Supplementary file 1 [file DataSheet1.docx]
